# Incidence of Coronary Artery Disease After Permanent Pacemaker Implantation: A Hospital-based Study from East India

**DOI:** 10.19102/icrm.2024.15065

**Published:** 2024-06-15

**Authors:** Dilip Kumar, Rabin Chakraborty, Siddhartha Goutam, Sanjeev S. Mukherjee, Debopriyo Mondal, Rana Rathor Roy, Ashesh Halder, Soumya Patra, Arindam Pande, Abhishek Roy, Suvradip Dutta

**Affiliations:** 1 Department of Interventional Cardiology, Medica Superspecialty Hospital, Kolkata, India; 2 Department of Pharmacology, IMS & SUM Hospital - II, Odisha, India

**Keywords:** Bradyarrhythmias, complete heart block, coronary artery disease, ejection fraction, pacemaker implantation

## Abstract

Bradyarrhythmias, characterized by heart rates of <60 bpm due to conduction issues, carry risks of sudden cardiac death and falls. Pacemaker implantation is a standard treatment, but the interplay between bradyarrhythmias, coronary artery disease (CAD), and patient attributes requires further exploration. This study was a retrospective hospital record–based study that analyzed data from 699 patients who underwent pacemaker implantation for symptomatic bradyarrhythmias between February 2019 and February 2022. Clinical parameters, coronary angiography (CAG) findings, ejection fraction, and indications for pacemaker implantation were documented. The relationship between CAD severity, specific bradyarrhythmias, and ejection fraction was explored. Statistical analysis included chi-squared tests and *t* tests. The mean age of the study population (n = 699) was 66.75 years (male:female ratio, 70:30), with 77.2% having type 2 diabetes and 61.6% being hypertensive. The majority of patients had minor or non-obstructive CAD (61.8%), followed by normal CAG findings (25.75%) and obstructive CAD (12.45%). Complete heart block (CHB) was the primary indication for pacemaker implantation (55.2%), followed by sick sinus syndrome (22.3%). The results did not show any association between ejection fraction and CAG findings. Patients who presented with CHB had a higher incidence of obstructive CAD, indicating greater severity. This study sheds light on the intricate interplay between severe bradyarrhythmias, CAD, and patient characteristics. Our analysis revealed no statistical significance between obstructive CAD and the need for a permanent pacemaker. This makes us question our practice of maintaining a low threshold for coronary angiography during pacemaker implantation. The observed low yield and anticoagulation protocol reassure us of the choice to delay this diagnostic intervention. These insights can guide tailored management strategies, enhancing clinical care approaches for patients with severe bradyarrhythmias necessitating pacemaker implantation.

## Introduction

Bradyarrhythmias are cardiac rhythms that occur at a rate of <60 bpm and are caused by conduction problems and automaticity disorders in the heart, such as sinus node disease or atrioventricular (AV) block.^1^ There is an increased risk of abrupt cardiac mortality due to heart slowing or stopping, as well as falls, particularly in elderly adults, due to fainting.

Every year, nearly one million pacemakers are implanted in the world to treat bradyarrhythmias.^2,3^ This is projected to rise as the population ages.^4^ New technologies, such as leadless pacemakers, are being introduced, which general practitioners may encounter more frequently. Such technological developments will assist physicians in properly identifying and managing bradyarrhythmias, as well as familiarizing them with an increasingly complicated assortment of pacemakers.

In adults, bradycardia has traditionally been defined by consensus as a slow heart rate (HR) of <60 bpm.^5^ This and other bradyarrhythmias can be caused by a variety of intrinsic and extrinsic factors that impair the conduction system. Furthermore, bradycardia can be a natural physiologic reaction in some situations (eg, during sleep or in healthy athletes). Bradyarrhythmias can present clinically as asymptomatic electrocardiographic (ECG) abnormalities or as a wide range of symptoms that can be typical (syncope, near-syncope) or atypical (dyspnea, angina, dizziness, weariness, lethargy).^6^

Symptoms can be persistent or periodic and inconsistent. Many people with conduction system disease are undiagnosed and never seek medical care.^7,8^ In terms of the etiology of bradyarrhythmias, it is difficult to attribute conduction system disorders to a single source in a significant proportion of individuals. Although many of these individuals actually have heart disease, usually ischemic, establishing a cause-and-effect relationship is difficult. In fact, unless the conduction defect occurred during an acute myocardial infarction, a histologic evaluation of the conduction system in the majority of these patients reveals non-specific scarring and fatty infiltration independent of the presence or absence of underlying ischemic heart disease.^9^

The study draws on hospital records to capitalize on their comprehensive clinical data, enabling a genuine representation of patient attributes and real-world severe bradyarrhythmia scenarios for insightful characterization. The primary objective of this study was to comprehensively describe the underlying characteristics of individuals with severe bradyarrhythmias necessitating pacemaker implantation, thereby enhancing our understanding of this patient population and their clinical profiles.

## Methodology

### Study design

This study adopts a retrospective hospital record-based design. Data were collected from patients who presented at our institute with symptomatic bradyarrhythmias necessitating primary permanent pacemaker implantation between February 2019 and February 2022. Ethical approval was obtained from the institutional ethics committee.

### Study population

A total of 1086 patients underwent permanent pacemaker insertion during the study period. These patients were subjected to specific inclusion and exclusion criteria, resulting in the inclusion of 699 patients for the study. Patients aged ≥40 years with symptomatic bradycardia meeting the American College of Cardiology (ACC)/American Heart Association (AHA)/Heart Rhythm Society (HRS) 2018 guidelines for permanent pacemaker insertion and providing consent for coronary angiography (CAG) prior to pacemaker insertion were included. Separately, those with missing or incomplete data and patients diagnosed with acute coronary syndrome were excluded.

### Data collection

Clinical parameters, demographic information, CAG findings, ejection fraction, and indications for pacemaker implantation were documented. The diagnosis of specific bradyarrhythmias (sinus node dysfunction, AV block, etc.) was based on 12-lead ECG findings or ECG evidence from Holter monitoring. Information regarding underlying cardiac conditions, blood pressure, fasting blood glucose, and lipid profile was collected. Conventional coronary risk factors such as sex, age, diabetes mellitus, and hypertension were recorded.

### Coronary angiography

CAG was conducted concurrently with pacemaker implantation or within 1 day prior. Multiple views of the left and right coronary arteries were taken. This study involved measuring the diameter and assessing stenosis severity and qualitative blood flow, particularly to territories supplying the conduction system. Luminal stenosis was quantified based on the visual estimation of diameter reduction in diseased segments compared to proximal disease-free reference segments. Significant coronary artery disease (CAD) was categorized as obstructive (≥50% stenosis) or non-obstructive (<50% stenosis). Stenosis severity was further classified as mild (50%–70%), moderate (70%–90%), or severe (>90%). Nodal arteries, such as the sinus nodal artery and AV nodal artery, were identified. Significant disease was defined as >50% stenosis in the nodal artery or its feeding artery proximal to the origin.

### Statistical analysis

SPSS version 20 (IBM Corp., Armonk, NY, USA) was used for statistical analysis. Patients were categorized based on CAG findings. Quantitative variables are presented as mean ± standard deviation values, and qualitative variables are presented as percentages. An independent-samples *t* test or chi-squared test was used for assessing parametric values between groups, as appropriate. The chi-squared test was also used for comparing categorical variables. Statistical significance was determined at *P* < .05 (two-tailed).

## Results

### Study population characteristics

A total of 699 hospital records were analyzed for subjects who underwent primary pacemaker implantation (PPI) due to underlying causes. The mean age of the study population was 66.75 ± 9.37 years (male, 67.18 ± 9.11 years; female, 65.76 ± 9.93 years), with a male preponderance (male:female ratio, 70:30). Type 2 diabetes was present in 77.2% of subjects, and 61.6% were hypertensive. The baseline parameters of the study subjects, along with CAG findings, are presented in **Table 1**.

### Causes of primary pacemaker implantation

Regarding CAD, most patients belonged to the minor obstruction group (minor CAD, 62.5%; normal CAG findings, 25.7%; obstructive CAD, 12.8%). The major cause of pacemaker implantation was complete heart block (CHB) (55.2%) followed by sick sinus syndrome (SSS) (22.3%), 2:1 AV block (6.5%), bifascicular block (6.7%), left bundle branch block (4.3%), and others (5%). The left anterior descending artery was observed in 52%, the right coronary artery (RCA) was observed in 25%, and the left circumflex artery (LCx) was observed in 6% of the study population, respectively. Furthermore, 17% of the patients had multivessel disease. The prevalence of atrial fibrillation was 38% among patients with SSS in the current study. Among cases involving the LCx, 80% of patients had a non-dominant vessel disease, while all cases involving the RCA had a dominant vessel disease. The relationship between CAG findings and the cause of PPI is shown in **Table 2**.

**Figure 1:**
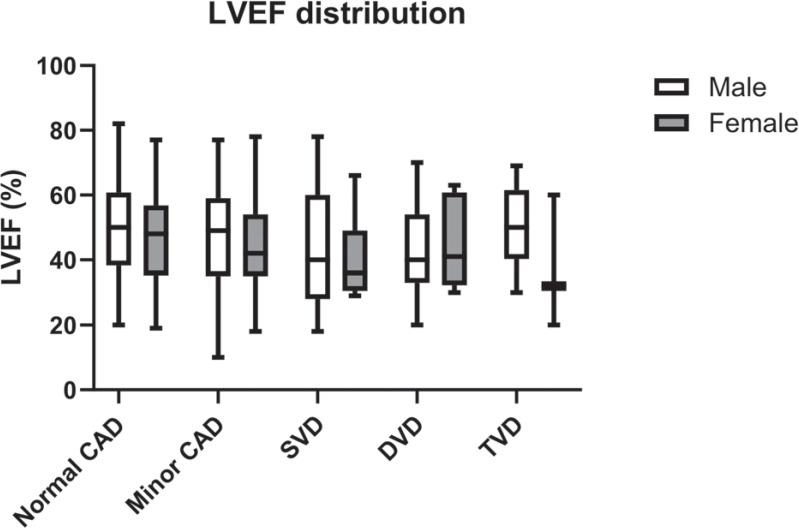
Median ejection fraction in different coronary angiography findings with respect to sex. *Abbreviations*: CAD, coronary artery disease; DVD, double-vessel disease; LVEF, left ventricular ejection fraction; SVD, single-vessel disease; TVD, triple-vessel disease.

The ejection fraction of these patients is demonstrated in various CAG findings with respect to sex in **Figure 1**.

**Figure 2:**
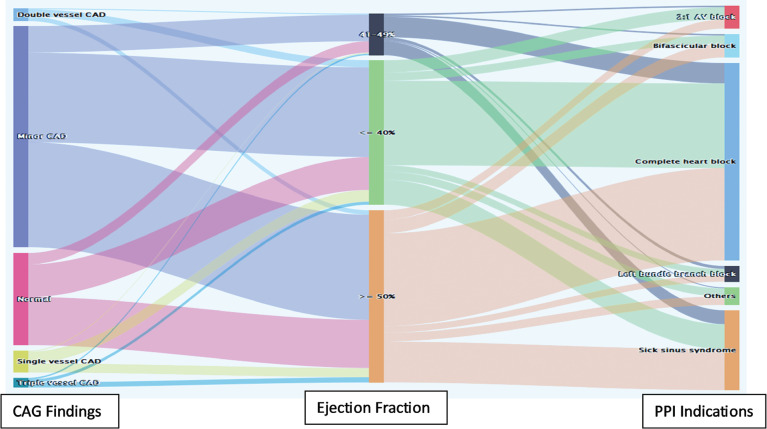
Comparing ejection fraction with coronary angiography findings and primary pacemaker implantation indications using the Sankey diagram. *Abbreviations:* CAG, coronary angiography; PPI, primary pacemaker implantation.

A Sankey diagram was plotted to draw attention to the major CAG findings in each category of ejection fraction (≤40%, 41%–49%, ≥50%) and the major PPI indicators observed in each ejection fraction category **(Figure 2)**.

## Discussion

Our study comprehensively describes the underlying characteristics of patients with severe bradyarrhythmias requiring pacemaker implantation. By investigating the interplay between severe bradyarrhythmias and CAD, we aimed to provide a nuanced understanding of the patient profiles and factors influencing the need for pacemaker intervention.

The study population’s demographic profile, with a mean age of 66.75 years, included a larger proportion of men (70%) than women (30%). These demographics are consistent with the documented trends in cardiovascular illness, where increasing age and male sex have been linked to a greater risk of heart problems.^10^ The prevalence of type 2 diabetes (77.2%) and hypertension (61.6%) highlights the interaction of chronic comorbidities with bradyarrhythmias, which may contribute to disease severity and management problems.^11^

The distribution of CAD findings based on angiography in our study revealed that the majority of patients (61.8%) had minor or non-obstructive CAD, followed by those with normal CAG findings (25.75%), and a smaller number (12.45%) had obstructive CAD. These proportions represent the complexities of CAD presentations in the setting of bradyarrhythmias requiring pacemaker implantation. Previous studies have observed incidences of obstructive CAD within the ranges of 13.7%–34.4% based on different definitions,^10,12,13^ which are higher than the findings of our current study (12.45%). This highlights the diverse nature of our patient group and underscores the importance of studying the interaction between bradyarrhythmias and CAD.^12^

CHB was found to be the most common cause of PPI (55.2%), followed by SSS (22.3%) and other arrhythmias. These findings are consistent with previous research^14^ that reported that patients with postoperative CHB can accurately be identified to undergo permanent pacemaker placement, emphasizing the critical significance of conduction system abnormalities in necessitating pacemaker intervention. The incidence of CHB and SSS emphasizes the therapeutic need for early detection and intervention to improve patient outcomes.^14^

Our research into the connection between CAD severity and specific PPI causes has yielded some intriguing results. While patients with obstructive CAD are a smaller cohort (6.6%), the proportion of CHB cases in this group is significant. This finding reveals that these patients require more clinical care than other subgroups. Furthermore, the disparity in the incidence of certain bradyarrhythmia subtypes across CAD categories highlights the complex interplay between cardiac anatomical abnormalities and conduction problems.^10^

In contrast to the study’s major focus on the relationship between CAD and PPI causes, we found no statistically significant associations between ejection fraction and CAG findings. While our data did not show that ejection fraction had a direct influence on the reasons for pacemaker implantation, it is nevertheless important to consider in a full patient evaluation. These findings support the hypothesis that cardiac function, as measured by ejection fraction, is an important predictor of overall cardiovascular health. Importantly, this information influences prognosis and informs therapy decisions, which is in accordance with prior research findings that conclude that CAD is a significant risk factor for incident heart failure with preserved ejection fraction, and this association is partly explained by echocardiographic changes, particularly in left ventricular diastolic function.^15^

The findings of this study hold several clinical implications. Understanding the prevalence of CAD in patients requiring PPI enhances risk stratification and treatment planning. Furthermore, the identification of specific bradyarrhythmias associated with obstructive CAD underscores the need for comprehensive cardiovascular assessment in these cases. These insights can guide physicians in devising tailored management strategies and selecting optimal therapeutic interventions.

### Limitations

The retrospective nature of this study warrants consideration of its limitations, including potential biases in data collection and incomplete records. The single-center design may limit the generalizability of findings. However, the comprehensive clinical dataset and adherence to ethical guidelines strengthen the reliability of the study’s outcomes.

## Conclusion

In conclusion, this study reveals the complex connection between severe bradyarrhythmias needing pacemaker implantation and CAD. Recognizing cardiac function is essential despite the lack of clear links between ejection fraction and CAD findings. In the area of severe bradyarrhythmias and their connection to CAD, this study emphasizes the significance of thorough patient evaluation and considers CAG when absolutely necessary to customize management techniques, providing insights that could ultimately improve clinical care approaches and patient outcomes.

## Figures and Tables

**Table 1:** Coronary Artery Disease Findings with Respect to Baseline Criteria of Patients

Parameters	Normal CAD (n = 180, 25.75%)	Minor or Non-obstructive CAD (n = 432, 61.8%)	Obstructive CAD (n = 87, 12.45%)	*P* Value
n	180	432	87	
Sex				
Male	100	320	70	<.00001
Female	80	112	17	
Age (years)				
<50	14	18	3	.37
50–75	140	343	69	
>75	26	71	15	
Hypertension				
Yes	111	262	58	.57
No	69	170	29	
T2DM				
Yes	142	328	70	.54
No	38	104	17	
LVEF				
≤40%	64	175	43	.27
41%–49%	22	52	7	
≥50%	94	205	37	

*Abbreviations:* CAD, coronary artery disease; LVEF, left ventricular ejection fraction; T2DM, type 2 diabetes mellitus.

**Table 2:** Causes of Primary Pacemaker Implant and Corresponding Coronary Angiography Findings

	Normal (n = 180)	Minor CAD (n = 432)	SVD (n = 43)	DVD (n = 25)	TVD (n = 19)
CHB					
Total	95 (52.8)	243 (56.3)	25 (58.1)	12 (48)	11 (57.9)
LAD			17 (39.5)	8 (32)	7 (36.8)
RCA			6 (13.9)	3 (12)	4 (21.1)
LCx			2 (4.7)	1 (4)	0 (0)
2:1 AV block					
Total	11 (6.1)	26 (6.0)	3 (7.0)	3 (12)	2 (10.5)
LAD			2 (4.7)	2 (8)	2 (10.5)
RCA			1 (2.3)	1 (4)	0 (0)
LCx			0 (0)	0 (0)	0 (0)
SSS					
Total	47 (26.1)	97 (22.5)	6 (13.9)	3 (12)	3 (15.8)
LAD			3 (7.0)	2 (8)	2 (10.5)
RCA			2 (4.7)	0 (0)	1 (5.3)
LCx			1 (2.3)	1 (4)	0 (0)
Bifascicular block					
Total	5 (2.8)	29 (6.7)	5 (11.6)	5 (20)	2 (10.5)
LAD			2 (4.7)	3 (12)	1 (5.3)
RCA			2 (4.7)	2 (8)	1 (5.3)
LCx			1 (2.3)	0 (0)	0 (0)
LBBB					
Total	15 (8.3)	15 (3.5)	0 (0)	1 (4)	0 (0)
LAD			0 (0)	1 (4)	0 (0)
RCA			0 (0)	0 (0)	0 (0)
LCx			0 (0)	0 (0)	0 (0)
Others					
Total	7 (3.9)	22 (5.1)	4 (9.3)	1 (4)	1 (5.3)
LAD			3 (7.0)	0 (0)	0 (0)
RCA			0 (0)	1 (4)	1 (5.3)
LCx			1 (2.3)	0 (0)	0 (0)

*Abbreviations:* CAG, coronary angiography; CHB, complete heart block; DVD, double vessel disease; LAD, left anterior descending artery; LBBB, left bundle branch block; LCx, left circumflex artery; RCA, right coronary artery; SSS, sick sinus syndrome; TVD, triple vessel disease. Patients with multivessel disease are a subset of DVD and TVD patients in whom large diagonal and/or ramus was involved.
